# Ligand Binding of PR-10 Proteins with a Particular Focus on the Bet v 1 Allergen Family

**DOI:** 10.1007/s11882-020-00918-4

**Published:** 2020-05-19

**Authors:** Lorenz Aglas, Wai Tuck Soh, Amin Kraiem, Mario Wenger, Hans Brandstetter, Fatima Ferreira

**Affiliations:** 1 Department of Biosciences, University of Salzburg, Hellbrunner Str. 34, A-5020 Salzburg, Austria; 2 Present Address: Laboratory of Immunochemistry, WPI Immunology Frontier Research Center, Osaka University, Suita, Japan

**Keywords:** PR-10, Allergens, Bet v 1, Ligand, Flavonoid, Cytokinin

## Abstract

**Purpose of Review:**

Pathogenesis-related class 10 (PR-10) proteins are highly conserved plant proteins, which are induced in response to abiotic and biotic stress factors. To date, no unique biological function could be assigned to them. Rather a more general role of PR-10 in plant development and defense mechanisms has been proposed. In addition, some PR-10 proteins act as allergens by triggering allergic symptoms in sensitized individuals. Regardless of the diversity of reported activities, all PR-10 proteins share a common fold characterized by a solvent-accessible hydrophobic cavity, which serves as a binding site for a myriad of small-molecule ligands, mostly phytohormones and flavonoids.

**Recent Findings:**

Most of available data relate to the ligand binding activity of allergenic PR-10, particularly for those belonging to Bet v 1 family of allergens. Bet v 1 and its homologues were shown to bind flavonoids with high affinity, but the specificity appears to differ between homologues from different species. The flavonoid Q3O-(Glc)-Gal was shown to specifically bind to hazelnut Cor a 1 but not to Bet v 1. Similarly, Q3OS bound only to the major isoform Bet v 1.0101 and not to other closely related isoforms. In contrast, Bet v 1 and hazelnut Cor a 1 showed very similar binding behavior towards other flavonoids such as quercetin, genistein, apigenin, daidzein, and resveratrol.

**Summary:**

Recent research findings highlighted the importance of more precise knowledge of ligand binding for understanding the functional diversification of PR-10 proteins.

## Introduction

Pathogenesis-related class 10 (PR-10) proteins comprise a unique class of highly conserved phytoproteins found in both monocotyledonous and dicotyledonous plants. PR-10 are primarily cytosolic proteins, constitutively expressed in several plant tissues including roots, stems, flowering compartments, fruits, and pollen grains from certain plant species. Their expression is upregulated upon abiotic and biotic stress conditions, such as invading pathogenic viruses, bacteria and fungi, cold, salinity, drought, oxidative stress, ultraviolet radiation, and physical wounding [1]. Hence, it has been proposed that PR-10 proteins have no unique function but play a more general role in plant development and defense mechanisms [2, 3].

**Fig. 1:**
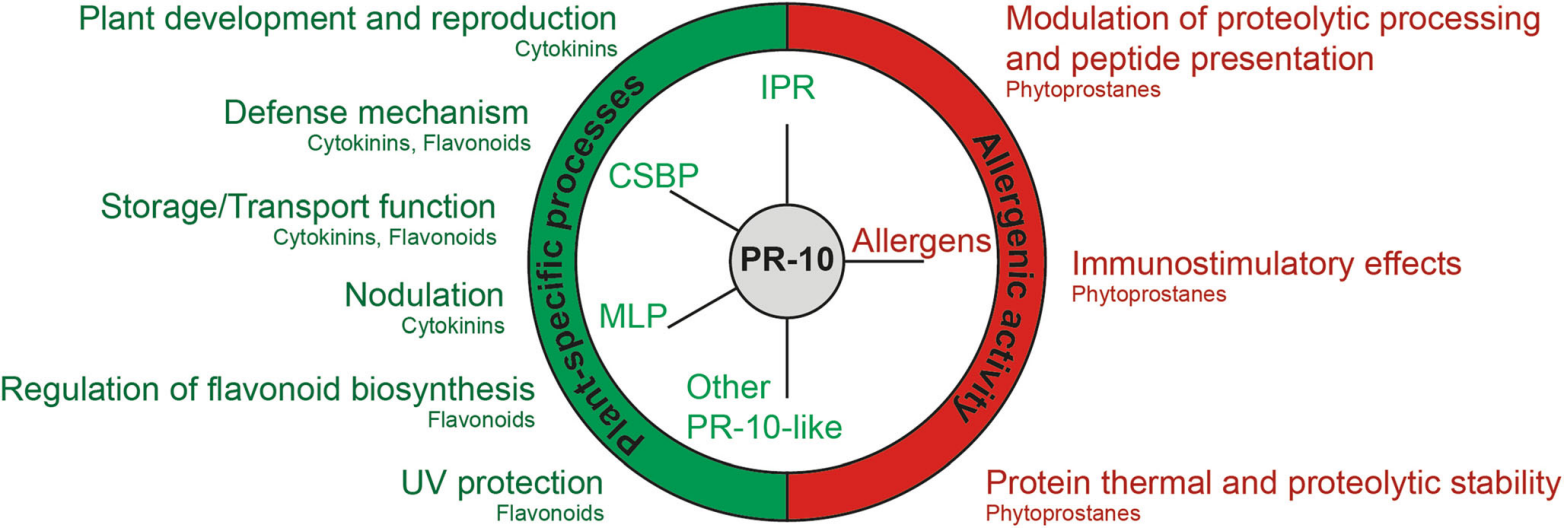
Overview on described functions associated with PR-10 ligand binding; PR-10, pathogenesis-related proteins class 10; IPR, intracellular pathogenesis-related proteins/classic PR-10 proteins; CSBP, cytokinin-specific binding proteins; MLP, major latex proteins; other PR-10-like, not yet classified proteins possessing a PR-10-like fold

PR-10 proteins are 154 to 163 amino acids long and have a molecular weight of approximately 17 kDa. Their 3-dimensional structure consists of an anti-parallel, seven-stranded β-sheets wrapping around an amphipathic C-terminal α helix (α3) embraced by two short α-helices (α1, α2) forming a V-shape. The main structural feature of the PR-10 fold is a large solvent-accessible, hydrophobic internal cavity spanning the entire protein [4, 5]. This hydrophobic core of PR-10 molecules serves as a binding site for a wide variety of ligands, thus explaining its promiscuous binding behavior. Here we review recent findings on ligand binding of PR-10 proteins with a particular focus on the Bet v 1 family and the impact of bound ligands on the immunological properties of these allergenic proteins. In addition, published data on PR-10 ligand binding in the broad context of plant biology are briefly discussed (Fig. 1).

## Ligand Binding of PR-10 Proteins

Ligand binding has been described for many PR-10 proteins, mostly for allergens derived from plants of the Fagales order, such as birch (*Betula verrucosa*), hazel (*Corylus avellana*), and beech (*Fagus sylvatica*). PR-10 proteins from more distantly related allergenic food sources like peanut (*Arachis hypogaea*), strawberry (*Fragaria ananassa*), cherry (*Prunus avium*), and peach (*Prunus persica*) were also shown to bind ligands [6••, 7••, 8–10]. Interestingly, not all reported ligand binding PR-10 proteins have been described as allergens. In fact, the taxon of *PR*-10 proteins can be subdivided into intracellular pathogenesis-related (IPR) proteins/classic PR-10, cytokinin-specific binding proteins (CSBP), and major latex proteins (MLP), although CSBP and MLP only share a sequence identity of about 17 to 25% with the IPR group [1]. In this review, we have additionally included “allergens” as another subgroup of PR-10 because not all proteins displaying the typical features and the canonical fold of classic PR-10 are allergenic. Nevertheless, their common ability to bind low molecular weight compounds within their hydrophobic cavity can be considered a general feature of PR-10 proteins. The promiscuous ligand binding behavior of PR-10 proteins has been extensively documented using a large panel of phytohormones and plant metabolites. Based on these studies, three major chemical classes of PR-10-binding ligands have been defined: (i) cytokinins, (ii) flavonoids, and (iii) sterols [10–13]. A detailed overview of PR-10 ligands described in the literature is given in Table 1.

**Table 1:** Protein-ligand interaction of PR-10 proteins

Protein	Plant (scientific name)	PR-10 class	Protein MW (kDa)	Ligand name	Ligand chemical class	Ligand MW (Da)	Kd (μM)	Method used to confirm ligand binding	Comment ^¤^	References
Ara h 8.0101	Peanut (*Arachis hypogaea*)	Allergens	16.95	Quercetin	Flavonoid	302.23	-*	ADA	-	[11]
				Genistein	Flavonoid	270.24	-*	ADA	-	[11]
				Apigenin	Flavonoid	270.24	-*	ADA	-	[11]
				Daidzein	Flavonoid	254.24	-*	Direct measurement	-	[11]
				Resveratrol	Flavonoid	228.24	-*	ADA	-	[11]
				Progesterone	Animal steroid hormone	314.50	-*	ADA	-	[11]
				Caffeic acid	Organic compounds	180.16	-*	ADA	-	[11]
				Palmitic acid	Flavonoid	256.42	-*	ADA	-	[11]
				Arachidic acid	Flavonoid	312.50	-*	ADA	-	[11]
Ara h 8.0201	Peanut (*Arachis hypogaea*)	Allergens	16.41	Quercetin	Flavonoid	302.23	-*	ADA	-	[11]
				Genistein	Flavonoid	270.24	-*	ADA	-	[11]
				Apigenin	Flavonoid	270.24	-*	ADA	-	[11]
				Daidzein	Flavonoid	254.24	-*	ADA	-	[11]
				Resveratrol	Flavonoid	228.24	-*	Direct measurement	-	[11]
				Progesterone	Animal steroid hormone	314.50	-*	ADA	-	[11]
				Caffeic acid	Organic compounds	180.16	-*	ADA	-	[11]
				Myristic acid	Flavonoid	228.37	-*	ADA	-	[11]
				Ferulic acid	Organic compounds	194.18	-*	ADA	-	[11]
Cor a 1.0201	Hazel pollen (*Corylus avellana*)	Allergens	17.41	Apigenin	Flavonoid	270.24	-*	ADA	-	[11]
				Daidzein	Flavonoid	254.24	-*	ADA	-	[11]
				Resveratrol	Flavonoid	228.24	-*	Direct measurement	-	[11]
				Progesterone	Animal steroid hormone	314.50	-*	ADA	-	[11]
				DOC	Brassinosteroid analog	414.60	-*	ADA	-	[11]
				Caffeic acid	Organic compounds	180.16	-*	ADA	-	[11]
				Myristic acid	Fatty acid	228.37	-*	ADA	-	[11]
				Palmitic acid	Fatty acid	256.42	-*	ADA	-	[11]
				Zeatin	Cytokinin	219.24	-*	ADA	-	[11]
				Quercetin	Flavonoid	302.23	-*	ADA	-	[11]
				Genistein	Flavonoid	270.24	-*	ADA	-	[11]
Cor a 1.0401	Hazelnut (*Corylus avellana*)	Allergens	17.58	Q3O-(Glc)-Gal	Flavonoid	626.53	Below 5	NMR	-	[7]
				Quercetin	Flavonoid	302.23	-*	ADA		[11]
				Genistein	Flavonoid	270.24	-*	ADA		[11]
				Apigenin	Flavonoid	270.24	-*	ADA		[11]
				Daidzein	Flavonoid	254.24	-^□^	ADA		[11]
				Epicatechin	Flavonoid	290.27	-^□^	ADA		[11]
				Resveratrol	Flavonoid	228.24	-*	Direct measurement		[11]
				Progesterone	Animal steroid hormone	314.50	-^□^	ADA		[11]
				DOC	Brassinosteroid analog	414.60	-^□^	ADA		[11]
				Caffeic acid	Organic compounds	180.16	-^□^	ADA		[11]
				Ferulic acid	Organic compounds	194.18	-^□^	ADA		[11]
				3-Indolybutyric acid	Organic compounds	203.24	-^□^	ADA		[11]
Fag s 1.0101	European beech (*Fagus sylvatica*)	Allergens	17.36	ANS	Organic compounds	299.30	-	ADA		[8]
				Dehydroergosterol	Brassinosteroid	394.60	-	ADA, NMR		[8]
				Naringenin	Flavonoid	272.25	-	ADA		[8]
				Kinetin	Cytokinin	215.21	-	ADA		[8]
				SDS	Synthetic detergent	288.38	-	ADA		[8]
Fra a 1.0101	Strawberry (*Fragaria ananassa*)	Allergens	17.77	Q3OS	Flavonoid	626.50	5.3	ITC	-	[10]
Fra a 1.0201				Myricetin (+)-catechin	Flavonoid	318.23	19.5	ITC	-	[10]
Fra a 1.0301					Flavonoid	290.27	8.9	X-ray, ITC	4C94, 1:2	[10]
Pru av 1.0101	Cherry (*Prunus avium*)	Allergens	17.66	Homocastasterone	Brassinosteroid	478.7	-	NMR, in silico docking	-	[14]
Pru p 1.0101	Peach (*Prunus persica*)	Allergens	17.65	Zeatin	Cytokinin	219.24	9.4	ITC	1:1	[9]
Que a 1.0201	White oak (*Quercus alba*)	Allergens	17.30	Daidzein	Flavonoid	254.24	-*	ADA	-	[11]
				Quercetin	Flavonoid	302.23	-*	ADA	-	[11]
				Genistein	Flavonoid	270.24	-*	ADA	-	[11]
				Apigenin	Flavonoid	270.24	-*	ADA	-	[11]
				Resveratrol	Flavonoid	228.24	-^□^	Direct measurement	-	[11]
				Progesterone	Animal steroid hormone	314.50	-^□^	ADA	-	[11]
				Palmitic acid	Fatty acid	256.42	-^□^	ADA	-	[11]
				Myristic acid	Fatty acid	228.37	-^□^	ADA	-	[11]
				DOC	Brassinosteroid analog	414.60	-^□^	ADA	-	[11]
Que a 1.0301	White oak (*Quercus alba*)	Allergens	17.48	Daidzein	Flavonoid	254.24	-*	ADA	-	[11]
				Quercetin	Flavonoid	302.23	-*	ADA	-	[11]
				Genistein	Flavonoid	270.24	-*	ADA	-	[11]
				Apigenin	Flavonoid	270.24	-*	ADA	-	[11]
				Resveratrol	Flavonoid	228.24	-^□^	Direct measurement	-	[11]
				Progesterone	Animal steroid hormone	314.50	-^□^	ADA	-	[11]
				Palmitic acid	Fatty acid	256.42	-^□^	ADA	-	[11]
BpPR-10c	European white birch (*Betula verrucosa*)	IPR	17.00	Kinetin	Cytokinin	215.21	-	NMR, in silico docking	1:1	[12]
				Hyperoside	Flavonoid	464.4	-	NMR, in silico docking	1:2	[12]
				Rutin	Flavonoid	610.5	-	NMR, in silico docking	1:1	[12]
				Emodin	Plant metabolite	270.24	-	NMR	-	[12]
				DOC	Brassinosteroid analog	414.60	-	NMR, in silico docking	1:2	[12]
LlPR-10.1A	Yellow Lupine (*Lupinus luteus*)	IPR	16.86	Zeatin	Cytokinin	219.24	-	In silico docking	2QIM, 1:3	[15]
LlPR-10.1B			16.66	Diphenylurea	Cytokinin	212.25	-	X-ray	3E85, 1:4	[16]
MtN13	Barrelclover (*Medicago truncatula*)	IPR	18.18	Zeatin	Cytokinin	219.24	-	X-ray	4JHG, 2:2	[13]
				Kinetin	Cytokinin	215.21	-	X-ray	4JHH, 2:3	[13]
				IPA	Cytokinin	203.24	-	X-ray	4GY9, 2:2	[13]
				N6-benzyladenine	Cytokinin	225.25	-	X-ray	4JHI, 2:2	[13]
SPE16	Jicama (*Pachyrhizus erosus*)	IPR	15.81	ANS	Organic compounds	299.30	-	X-ray	1TXC, 2:5	[1]
UBP34	Spreading earthmoss (*Physcomitrell a patens*)	IPR	34.00	azido-CPPU	Cytokinin agonist	288.69	25	Direct measurement	-	[17]
VrCSBP	Mung bean (*Vigna radiata*)	CSBP	17.60	Zeatin	Cytokinin	219.24	106–161	ADA, ITC, X-ray	2FLH, 1:1	[18, 19]
				ANS	Organic compounds	299.30	32.5	Direct measurement	-	[18, 19]
				4PU30	Cytokinin	247.68	1.3	ADA	1:1	[18, 19]
				IPA	Cytokinin metabolite/zeatin precursor	203.24	11.5	ADA	1:1	[18, 19]
				Kinetin	Cytokinin	215.21	64.1	ADA	1:1	[18, 19]
NCS	Yellow meadow-rue (*Thalictrum flavum*)	Others	23.34	4-Hydroxybenzaldehyde Dopamine	Plant metabolite	122.12	-	X-ray	2VQ5, 2:1	[20]
					Catecholamine	153.18	-	X-ray	2VQ5, 2:2	[20]
Hyp-1	Saint John’s wort (*Hypericum perforatum*)	Others	17.81	Melatonin ANS	Biogenic amine, hormone	232.28	-	X-ray	3IE5, 1:3	[21]
					Organic compounds	299.30	-	X-ray	4N3E, 1:4	[21]

^¤^3D structure (PDB code) and binding stoichiometry (Protein:Ligand); *probably strong binding; ^□^probably weak binding

*ADA*, ANS displacement assay; *Allergens,* characterized allergens according to the WHO/IUIS allergen nomenclature database; *CSBP*, cytokinin-specific binding proteins; direct measurement, direct measurement by florescence of ligand molecule; *IPR*, intracellular pathogenesis-related proteins/classic PR-10; *ITC*, isothermal titration calorimetry; *Kd*, equilibrium dissociation constant; *MW*, molecular weight; *NMR*, nuclear magnetic resonance spectroscopy; *others*, not yet classified proteins possessing a PR-10-like fold; *X-ray*, X-ray crystallography

Cytokinins are phytohormones involved in the regulation of plant development, growth and defense mechanisms, cell division, and the deceleration of senescence [9]. Binding to the naturally occurring cytokinins zeatin and kinetin has been mostly described for CSBPs [8, 9, 12–18, 20–23, 24••]. According to the current hypothesis, CSBPs might have evolved within PR-10 proteins in order to maintain cytokinin homeostasis by specifically binding to these molecules. Thus, the specific PR-10 fold is highly conserved within the CSBP group supporting the notion that their fold enables efficient shuttling and storage of bioactive molecules [18, 19]. Interestingly, the PR-10 from *Medicago truncatula* (MtN13) was observed to play a role as regulator of free cytokinins during the early phases of nodulation, i.e., the recruitment process of symbiotic nitrogen-fixing bacteria [13]. Based on the fact that zeatin is a nucleoside analog, PR-10 proteins have been proposed to possess nuclease activity [15]. In this respect, several studies focusing on the putative RNase or DNase activity of PR-10 proteins derived from several plant species including birch, cotton, ginseng, peach, and pepper have been reported. However, the biological relevance of these studies has not been further explored and the reported PR-10/DNA/RNA interactions are still a matter of debate [9, 12, 15, 25–30]. Apart from the cytokinins zeatin and kinetin, other phytohormones, such as phytomelatonin, brassinosteroids, and gibberellic acid, were frequently observed to bind PR-10 proteins [14, 31].

Another important class of PR-10 ligands are flavonoids, which are polyphenolic compounds and secondary plant metabolites involved in color and flavor production, UV protection, antioxidation, and pathogen defense [32]. PR-10 allergens, mostly of the Fagales order, were shown to bind flavonoids with high binding affinity within their hydrophobic pocket, but the specificity appears to differ between PR-10 from different species. For instance, the natural ligand of hazelnut Cor a 1, Q3O-(Glc)-Gal is highly similar to the birch Bet v 1 co-purified natural ligand, quercetin-3-O-sophoroside (Q3OS), differing just in the orientation of the hydroxyl group. Nevertheless, the Cor a 1 ligand does not interact with Bet v 1 and vice versa, despite the high structural and sequence similarity between both allergens [7••]. In addition, Q3OS bound only to the major isoform, Bet v 1.0101 and not to other isoforms [33]. These observations are in line with the notion that the presence of many PR-10 isoforms in a plant could be the basis for their functional diversification [34]. In contrast, a study by Mc Bride et al. reported a more general, less discriminative binding of Bet v 1 and hazelnut Cor a 1 to other flavonoids (e.g., quercetin, genistein, apigenin, daidzein, resveratrol), highlighting the relative binding promiscuity of PR-10 allergens for this class of ligand [11]. Accordingly, other PR-10 allergens, such as strawberry Fra a 1, white oak Que. a 1, hazel pollen Cor a 1, and peanut Ara h 8, also bound numerous flavonoids [10, 11, 32]. The exact role of flavonoid binding by PR-10s remains to be elucidated; however, it is speculated that this protein class possesses major regulatory functions in flavonoid biosynthesis via binding and storage of functionally inert glycosylated flavonoids, which are in turn prevented from early activation by enzymatic deglycosylation by glycosyltransferases [7••, 10, 32]. This hypothesis is supported by the observation that the major allergen of beech pollen, Fag s 1, binds with high affinity to naringenin, a metabolic intermediate of flavonoid synthesis, whereas it does not interact with unglycosylated quercetin [8]. Similarly, the binding affinity of Bet v 1 to Q3OS was 60-fold stronger compared with quercetin [4].

## Ligand Binding of Bet v 1

IgE sensitization to Bet v 1, the major allergen of birch (*Betula verrucosa*) pollen, ranges from 53 to 95% among birch pollen allergic patients. Similar sensitization rates were observed for Bet v 1 homologues like Mal d 1 (apple) and Cor a 1 (hazelnut), mostly due to cross-reactivity occurring between structurally similar PR-10 proteins [35, 36]. This results in clinical manifestations described as the oral allergy syndrome (OAS), a very common allergic disorder affecting more than 50% of birch pollen allergic patients [37]. Bet v 1.0101 (formerly designated Bet v 1a) represents the most abundant isoform of Bet v 1 comprising 50 to 70% of the allergen in pollen [4, 5]. The natural function of Bet v 1 is still not fully understood; however, based on the structural similarity with the START domain of the human MLN64 protein, it has been suggested that Bet v 1 play a role in steroid binding [38]. The ability of Bet v 1 to bind a broad spectrum of plant intrinsic ligands, such as fatty acids, cytokinins, or flavonoids, has led to the suggestion of an involvement in different stages of plant reproduction (e.g., protection of pollen DNA from UV-damage, transportation of lipids or flavonoids to the stigmatic surface to support pollen hydration and germination). Bet v 1 may also act as an storage scaffold for such ligands, enabling their rapid release upon seed germination [39]. In addition, the combination of different ligands and Bet v 1 isoforms with differences in their ligand preferences could possibly serve as molecular fingerprints to prevent self-pollination [23, 33, 40].

Bet v 1 has been shown to bind a broad range of hydrophobic to amphipathic ligands, differing in size and shape, to distinct binding sites within its hydrophobic cavity [23]. In general, pollen-derived molecules that interact with Bet v 1 can be grouped into fatty acids, flavonoids, and phytohormones. A detailed description of Bet v 1 ligands reported in the literature is given in Table 2.

**Table 2:** Ligand binding of Bet v 1

Chemical class	Ligand name	MW (Da)	Kd (μM)	3D structure	(PDB method used to confirm ligand binding code)	Stoichiometry (Protein:Ligand)	References
Brassinosteroids	24-Epicastasteron	464.70	**-**	**-**	MS	1:1	[41]
	Brassinolide	480.70	**-**	**-**	MS, in silico docking studies	1:1 and 1:2	[41]
	Dehydroergosterol	394.60	**-**	**-**	ADA	-	[23]
	Stigmasterol	412.70	**-**	**-**	ADA	-	[11]
Cytokinins	IPA	203.24	64.3	-	ADA	-	[23]
	Kinetin	215.21	84.1	4A85, 4A86	ADA, X-ray	1:1	[23, 42]
	Zeatin	219.24	-	-	ADA	-	[11]
Phytoprostanes	Phytoprostane B1	308.40	1		SAW		[6••]
	Phytoprostane E1	356.50	0.5		NMR, SAW		[6••]
	Phytoprostane F1	328.40	2.4		SAW		[6••]
Fatty acids	Arachidic acid	312.50	26.9		ADA		[23]
	Myristic acid	228.37	8.7		ADA		[23]
	Palmitic acid	256.42	7.7		ADA		[23]
	Stearic acid	284.50	4.2		ADA		[23]
Flavonoids	Apigenin	270.24	-	-	ADA	-	[11]
	Daidzein	254.24	-	-	ADA	-	[11]
	Flavone	222.24	33.2	-	ADA	-	[23]
	Genistein	270.24	-	-	ADA	-	[11]
	Naringenin	272.25	28.6	4A87	ADA, X-ray	1:1	[23, 42]
	Q3OS	626.50	0.56 to 1.5	-	NMR, MS, SAW	-	[4, 6••]
	Quercetin	302.23	9.2 to 33	-	NMR, ADA	-	[4, 23]
	Resveratrol	228.24	-	-	ADA	-	[11]
Extrinsic and non-physiological ligands	ANS	299.30	18.5	4A80, 4A8V, 4A86	Direct measurement, NMR, X-ray	1:1	[23, 42]
	DOC	414.60	58.8	1FM4, 4A81, 4A83	X-ray, MS	1:2	[6••, 41, 42]
	NDSB-256	257.35	-	4A8G	ADA, X-ray	1:1	[42]
	P303	304.06	-	4MNS	X-ray	1:1	[43]
	Progesterone	314.50	-	-	ADA	-	[11]
	SDS	288.38	7* and 100^□^	4QIP	ADA, X-ray	1:2	[23, 44]
	STS	316.43	1* and 20^□^	-	NMR	1:2	[44]

*Inner binding site; ^□^outer binding site

*ADA*, ANS displacement assay; direct measurement, direct measurement by florescence of ligand molecule; *Kd*, equilibrium dissociation constant; *MS*, mass spectrometry; *MW*, molecular weight; *NMR*, nuclear magnetic resonance spectroscopy; *SAW*, surface acoustic wave measurement; *X-ray*, X-ray crystallography

The affinity of Bet v 1 towards fatty acids depends on their chain length and reaches a maximum at 14 to 18 carbon atoms including stearate, palmitate, and myristate. Among flavonoids, flavone, naringenin, apigenin, genistein, quercetin, and daidzein were shown to interact with Bet v 1. The cytokinins N6-(2-isopentenyl)adenine (IPA), kinetin, and zeatin bind to Bet v 1 with lower affinities, whereas the phytohormones indole-3-acetic acid, gibberellic acid, and abscisic acid showed no interaction [11, 23].

Other phytohormones reported to bind Bet v 1 in vitro are the brassinosteroids, brassinolide, and 24-epicastasteron [41]. However, the interaction was analyzed using Bet v 1.0107 (formerly designated Bet v 1l), a hypoallergenic isoform constituting only up to 7% of the total Bet v 1 content in birch pollen [45, 46]. Dehydroergosterol, a frequently used cholesterol model compound, as well as progesterone and the plant sterol stigmasterol were demonstrated to bind Bet v 1 [11, 23]. Recently, phytoprostane E1 (PPE1) and its derivatives B1 (PPB1) and F1 (PPF1) were shown to bind to Bet v 1.0101 with dissociation constants (Kds) of 0.5, 1.0, and 2.4 μM, indicating high binding affinity [6••]. Phytprostanes were detected in relevant quantities in birch pollen and shown to be associated with the induction of immune responses relevant for allergic sensitization [47, 48].

In 2014, Seutter von Loetzen et al. isolated the glycosylated flavonoid Q3OS in complex with Bet v 1 directly from birch pollen extracts. Its binding to this PR-10 protein was confirmed using nuclear magnetic resonance (NMR) spectroscopy and surface acoustic wave (SAW) technology, and is now considered a physiological ligand of Bet v 1 [4, 6••]. Q3OS is a catechol derivative able of binding iron ions [49]. In vitro, the presence of iron strongly affected the affinity of Q3OS to Bet v 1, potentially impacting its allergenicity [50].

Bacterial toll-like receptor (TLR) agonists were often suggested as ligands of Bet v 1 able to modulate its allergenicity [51, 52]. However, this notion has been challenged by a recent study from our laboratory in which very high dissociation constants were measured for the TLR4 agonist lipopolysaccharide (LPS) and the TLR2 agonist lipoteichoic acid (LTA) (199.8 and 185.0 μM, respectively), indicating no physiologically relevant binding [6••].

Besides plant-derived ligands, several artificial model ligands and extrinsic molecules binding Bet v 1 were investigated including the bile acid sodium deoxycholate (DOC), a structural analog of brassinosteroids able to bind Bet v 1 at two distinct sites [6••, 41–43, 53]. Other non-physiological ligands include the detergents sodium dodecyl sulfate (SDS), sodium tetradecyl sulfate (STS), and 3-[Benzyl(dimethyl)ammonio]propane-1-sulfonate (NDSB-256) [6••, 23, 42, 44]. Studies using these surrogate compounds mainly focused on the effects (e.g., fold stability, dynamics, conformation) induced by ligand binding.

## Influences on Physicochemical Properties and Proteolytic Processing of Bet v 1

Several ligand binding-induced effects on the physicochemical properties of Bet v 1 were reported with a focus on the influences on the protein’s thermal stability and proteolytic susceptibility [6••, 53]. In general, ligand binding was observed to be associated with rigidification and compactness of the 3D structure of Bet v 1, while the conformational dynamics and flexibility were reduced. No significant alterations regarding the secondary structural element composition due to ligand binding were observed [6••]. Changes in structural dynamics of Bet v 1 affect the accessibility of its proteolytic cleavage sites to lysosomal proteases leading to a reduction in cleavage efficiency and consequently affecting the availability of Bet v 1 peptides for optimal peptide presentation to T cells via the major histocompatibility complex class II (MHC-II) receptor [54, 55]. Antigen presentation facilitated by antigen presenting cells is a necessary aspect for Th2 polarization, a key mechanism mandatory for the development of IgE-mediated allergic immune response. The presentation of allergen-derived peptides via the MHC-II pathway requires the processing of the allergen by lysosomal proteases. The influence of Bet v 1 fold stability on antigen presentation was elucidated by studying several fold stabilized mutants of Bet v 1. In comparison with wildtype Bet v 1, a mutant with optimal proteolytic stability to lysosomal proteases was able to stimulate a pronounced allergy-associated Th2 response in mice, suggesting that fold stability is a contributing factor for allergenicity [56].

In our recent study, describing a higher proteolytic resistance of Bet v 1 due to ligand binding, we have provided a mechanistic explanation for the increased allergenicity of the stabilized Bet v 1 mutant. The study also revealed a newly identified high affinity Bet v 1 ligand known as PPE1, which not only stabilized Bet v 1 from proteolytic degradation by lysosomal proteases but also modulated the proteolytic lysosomal activity of cysteine cathepsins [6••]. This dual-role of the ligand consequently influenced the presentation of the immunodominant Bet v 1 T cell epitope via MHC-II loading. Hence, ligand binding can regulate the processing of Bet v 1 by lysosomal proteases subsequently affecting availability of peptides for MHC-II presentation due to alterations in the frequency of proteolytic processing cleavage sites.

## Methods Used to Determine Ligand Binding

The ANS displacement assay (ADA) for assessing the binding of various hydrophobic ligands to Bet v 1 was first described by Mogensen et al. [23]. The assay is based on the ability of the fluorophore 8-anilino-1-naphthalenesulfonic acid (ANS) to interact with PR-10 proteins. ANS behaves as a weak fluorophore in solution, but upon binding to the hydrophobic patches of a protein, its fluorescence intensity dramatically increases [18]. ANS binds to Bet v 1 via its extended hydrophobic surface patches within its prominent cavity [42]. If the ANS binding sites are already occupied with other ligands, this results in a decrease of ANS fluorescence; thus, ligand-induced ANS displacement can be fluorescently monitored. Due to its convenience, ADA is usually the first method of choice to investigate ligand binding of PR-10 proteins, as indicated in Tables 1 and 2. However, the assay has the disadvantage of providing only an indirect measurement that relies on the capacity of ligands to share the same binding sites and to displace ANS. The analysis of the observed fluorescence changes can be complicated by several factors, such as inner filter effects or fluorescence of by the competing ligand. In the first case, a decrease would artificially indicate binding of the tested ligand where the decrease was mostly due to a reabsorption of the ANS fluorescence signal; in the second case, ligand binding could increase the fluorescence signal. Additionally, ligand interaction with ANS and/or Bet v 1 may tune the emission maximum of ANS, e.g., by inducing structural rearrangements which may also result in additional ANS interaction sites in the respective PR-10 molecule. Similarly to ANS, the naturally occurring polyphenol resveratrol was also observed to increase its intrinsic fluorescence upon binding to the hydrophobic patches of Bet v 1 [11].

Alternatives to ADA for the investigation of PR-10 ligand binding include direct measuring techniques, such as isothermal titration calorimetry (ITC), surface acoustic wave (SAW), and microscale thermophoresis (MST), which enable accurate determination of binding affinity and Kd [6••, 9, 18].

NMR and X-ray crystallography also proved to be powerful tools for the identification of structural rearrangements induced by ligand binding compared with the apo-protein, and for information on the binding stoichiometry [4, 6••, 7••, 42]. Both techniques can be used to precisely map the amino acid residues involved in ligand interaction at the respective binding sites. Interestingly, attempts to crystallize some PR-10 proteins without ligand were not successful, consistent with a superordinate role of ligands in protein rigidification and reduction of structural dynamics necessary for successful crystallization [13]. Besides the aforementioned techniques, in silico docking experiments were performed in several studies to evaluate the binding of ligands [15].

## Conclusions

Recent studies emphasize that PR-10 proteins’ structural and functional properties cannot be understood by their proteinogenic properties only. A structurally diverse spectrum of hydrophobic ligands can bind selectively to the PR-10 protein family, thereby tuning their physiologic and immunologic functions. Importantly, the organic cargo of PR-10 protein can exhibit secondary functions by addressing distant protein targets. The dual functions of protein ligands are exemplified by the phytoprostane PPE1, which affects protein stability of Bet v 1 and at the same time covalently blocks papain-related cysteine proteases via a Michael reaction warhead.

While the dual proteinogenic and organic composition shapes the physiological functions of PR-10 proteins, it as much accounts for their allergenic properties. The selective binding of ligands provides a rational for hypo- versus hyper-allergenic properties of allergen isoforms, which were previously explained by their differences in amino acid composition and 3d structure. In the light of the new findings, it may become possible to convert an allergen into a hypoallergen by differential ligand loading.

## Abbreviations

ANS: 8-Anilinonapthalene-1-sulfonic acid
CSBP: Cytokinin-specific binding proteins
DOC: Sodium deoxycholate
IPR: Intracellular pathogenesis-related proteins
Kd: Equilibrium dissociation constant
LPS: Lipopolysaccharide
LTA: Lipoteichoic acid
MHC II: Major histocompatibility complex class II
MLP: Major latex proteins
IPA: N6-(2-isopentenyl)adenine
MW: Molecular weight
NDSB-256: Dimethylbenzylammonium propane sulfonate
NMR: Nuclear magnetic resonance
PPA1, PPB1, PPE1, PPF1: Phytoprostane A1, B1, E1, and F1
PR-10: Pathogenesis-related proteins class 10
Q3OS: Quercetin 3-O-sophoroside
SAW: Surface acoustic wave
SDS: Sodium dodecyl sulfate
STS: Sodium tetradecyl sulfate
TLR: Toll-like receptor
